# Editorial: Exploration of Underutilized Food Sources and By-products to Reduce Food Losses and Waste

**DOI:** 10.3389/fnut.2021.751788

**Published:** 2021-11-30

**Authors:** Efigenia Montalvo-González, Juliana Morales-Castro, José M. Gil

**Affiliations:** ^1^Instituto Tecnológico de Tepic, Tepic, Mexico; ^2^Departamento de Ingeniería Química y Bioquímica, TecNM/Instituto Tecnológico de Durango, Durango, Mexico; ^3^CREDA-UPC-IRTA, Barcelona, Spain

**Keywords:** by-products, food losses, food waste, valorization, food residues

The demand for food increases with population growth and an inefficient food system. As one-third, 33% of produced food is lost as waste (1), mitigation strategies and actions are required. FAO estimates for the Food Loss Index, which calculates food losses only, not including the retail level, represents 14% of food production at the global level (2). At the same time, food insecurity is rising and efforts are being made to meet the needs of millions of people in need.

Food losses and food waste are complex multi-dimensional problems. In addition, upon disposal, food waste represents lost natural resources used for food production, loss in investment, and a negative impact on the environment. Some food by-products, such as inedible food, results from food processing industries such as plant-based, seafood, and beef residues.

Agricultural by-products have been traditionally used for animal feed, production of energy, and fuel, although by-products present some drawbacks such as lack of homogeneity due to different types and origin, biological instability, high Chemical Oxygen Demand (COD), and Biochemical Oxygen Demand (BOD). The heterogeneous chemical composition of by-products has catalyzed numerous studies to explore the potential of bioactive compounds for other sectors, such as the pharmaceutical, cosmetic, and nutraceutical industries (3).

In this way, food processing residues, as rich sources of food ingredients with nutritional, biological, and technological functionalities, represent potential sources of bioactive compounds and are available at low cost via emerging, efficient, extraction technologies (4). In addition, there are food sources that are under-exploited or that fall into disuse and are now being considered as a source of nutritional and bioactive components.

Circular Economy promotes upcycling and reduction of food waste. Food is designed to be cycled, keeping food in the value-circle as much as possible. Thus, valorization of food by-products is strongly encouraged where waste does not exist and is the feedstock for another cycle (5). Therefore, it is important to share and give visibility to research efforts to valorize by-products and underutilized foods, the theme of this special issue and covered by six articles:

An overview of the utilization of vegetable and fruit by-products as ingredients in line with circular economy principles, due to their nutritional value and ability to meet the demand for functional foods, is presented (Lau et al.); in addition to the nutritional value and health benefits, examples of food applications are covered.

Since production, the utilization of discarded food parts and by-products is sought through different post-harvest treatments by Caltagirone et al. Almond by-products, such as the skin, were separated from the seed using two methods, extracting important bioactive substances such as phenols and aromas.

Similarly, the valorization of by-products from *Prunus serotina* almond to obtain cold-pressed oil, such as from the shell and paste, results in the recovery of phenolics and flavonoids with high antioxidant potential and by-products with promising alternatives from a neglected product (Gallardo-Rivera et al.).

Despite the more than 400,000 plant species on earth, humans consume only about 200 species. And more incredibly, the typical human diet is based primarily on three crops, maize, rice, and wheat. Therefore, there are many food crops with reduced economic value that could be a potential source of nourishment and health benefits. Tepary Beans (*Phaseolus acutifolius* Gray) is an underutilized, native crop, with a low commercial value from the northern part of Mexico. The beans are used to prepare the flour and a protein concentrate and exhibit good bio-and techno-functional properties (López-Ibarra et al.).

Food wastes are a large concern due to the disposal costs of using landfills, which requires collection and transportation, and the generation of additional greenhouses gases (GHG) during decomposition. In this manner, alternative food use for food residues such as corn cob, generated after the grain is separated from sweet corn, are needed. One potential use is the utilization of cob flour to prepare dressing-type emulsions that were evaluated resulting in a promising additive to improve dressings (Castillo et al.).

Regarding seafood, the losses due to the processing of crabmeat are diminished when cooked crab meat is subjected to an additional processing step with enzymes plus cooking. Processing resulted in a restructured gel with better mechanical properties (Trejo-Díaz et al.).

The research articles published in this Research Topic contribute to the dissemination of novel strategies to reduce food losses and waste generated by the food industry. However, much more research is needed in the future, with the support of the government, industry, and the general public, to counteract the huge problem of food loss and waste.

## Author Contributions

EM-G, JM-C, and JG wrote and revised the manuscript. All authors contributed to the article and approved the submitted version.

## Conflict of Interest

The authors declare that the research was conducted in the absence of any commercial or financial relationships that could be construed as a potential conflict of interest.

## Publisher's Note

All claims expressed in this article are solely those of the authors and do not necessarily represent those of their affiliated organizations, or those of the publisher, the editors and the reviewers. Any product that may be evaluated in this article, or claim that may be made by its manufacturer, is not guaranteed or endorsed by the publisher.
